# Dermoscopy of Rippled Pattern Sebaceoma

**DOI:** 10.1155/2010/140486

**Published:** 2010-07-27

**Authors:** Mizuho Nomura, Masaru Tanaka, Maki Nunomura, Miki Izumi, Fuyuki Oryu

**Affiliations:** ^1^Departments of Dermatology, Tokyo Woman's Medical University Medical Center East, 2-1-10 Nishi-Ogu, Arakawa-ku, Tokyo 116-8567, Japan; ^2^Tachikawa-Sougo Hospital, Tokyo Woman's Medical University Medical Center East, 2-1-10 Nishi-Ogu, Arakawa-ku, Tokyo 116-8567, Japan; ^3^Departments of Diagnostic Pathology, Tokyo Medical University, Tokyo, Japan; ^4^Tachikawa-Sougo Hospital, Tokyo Medical University, Japan

## Abstract

A 77-year-old Japanese woman presented a dome-shaped pinkish nodule on the scalp. Dermoscopy demonstrated yellowish homogeneous ovoid areas with translucent whitish veil and arborizing vessels. No association with Muir-Torre syndrome was found. Histopathology revealed a smooth-bordered neoplasm in the dermis with partial connection to the epidermis. The tumor was composed mainly of germinative cells. The tumor focally showed a typical “rippled pattern”. There were only a few vacuolated cells suggesting sebaceous differentiation. These cells were highlighted with adipophilin antibody. No nuclear atypia or mitotic figures were observed. We regarded the neoplasm as sebaceoma. Dermoscopy demonstrated clearly visualized yellowish homogeneous ovoid areas. This feature usually corresponds to dermal conglomerations of the cells with sebaceous differentiation. However, this case histopathologically showed only limited area with sebaceous differentiation. We presented a case of rippled-pattern sebaceoma and described its dermoscopic features. This was the first report referring to the dermoscopic features of sebaceoma.

## 1. Case Report

**Figure 1:**
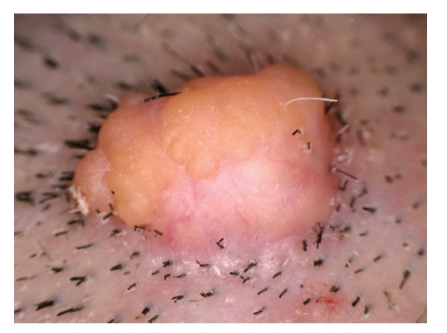
A dome-shaped faintly pinkish nodule. The surface of the tumor was covered with yellow papules.

**Figure 2:**
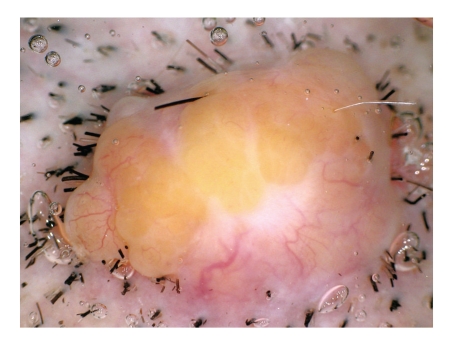
Yellowish homogeneous ovoid areas and arborizing vessels at the periphery of the nodule.

**Figure 3:**
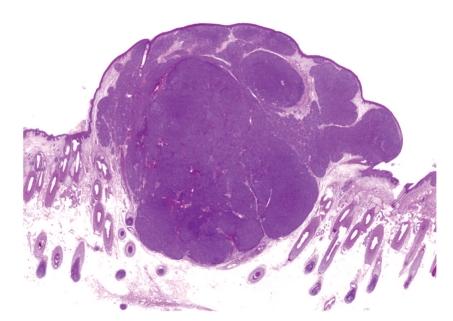
A well-circumscribed, smooth-bordered, and deeply basophilic tumor in the dermis.

**Figure 4:**
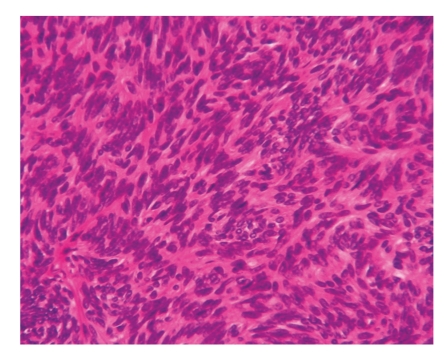
The tumor is composed of basaloid cells with deeply basophilic oval nuclei showing a typical rippled pattern.

**Figure 5:**
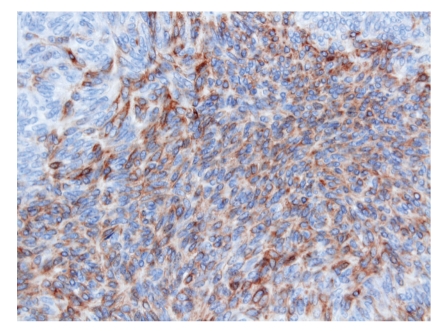
The cells simulating Verocay bodies are positive for AE1/AE3.

**Figure 6:**
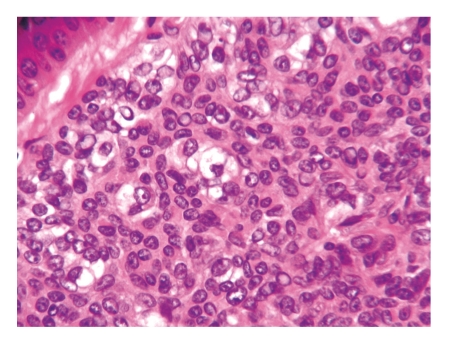
A few sebocytes are seen at the periphery of the tumor nests.

**Figure 7:**
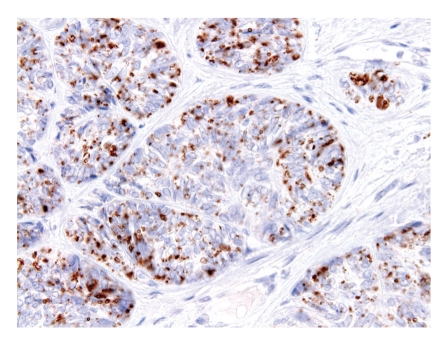
Sebocytes seen at the periphery of the tumor nests are positive with adipophilin.

A 77-year-old Japanese woman presented with a tumor on the parietal region of the scalp, which had gradually enlarged over the previous several years. Physical examination revealed a dome-shaped faintly pinkish nodule, 10 × 8 mm in size. The surface of the tumor was covered with yellow papules (Figure 1). Dermoscopic examination demonstrated yellowish homogeneous ovoid areas covered with translucent whitish veil and arborizing vessels at the peripheral peach-colored area of the nodule (Figure 2). The patient had no significant family or past history. No association with Muir-Torre syndrome was found. The lesion was suspected as being a sebaceous neoplasm and totally excised. Histopathological examination of the excised nodule revealed a well-circumscribed and smooth-bordered neoplasm in the entire dermis with partial connection to the epidermis (Figure 3). The tumor was multinodular, and the most part of the nodule was composed of germinative cells. In addition, the tumor focally showed a typical “rippled pattern” (Figure 4). The cells were arranged in linear rows parallel to one another, simulating Verocay bodies, which were positive for AE1/AE3 (Figure 5) but negative for S-100 protein. There were only a few vacuolated cells with foamy and bubbly cytoplasm, suggesting sebaceous differentiation especially at the superficial area of the region (Figure 6). These cells possess lipid vacuoles which were highlighted with adipophilin antibody (Figure 7). No nuclear atypia or mitotic figures were observed in the constituents of neoplastic cells. There were no features suggesting the existence of nevus sebaceus, such as sebaceous hyperplasia or ectopic apocrine glands around the tumor.

## 2. Discussion

Sebaceoma, originally described by Troy and Ackerman [1], is a distinct benign sebaceous neoplasm that is histopathologically characterized by dermal aggregations of sebaceous germinative cells and sebaceous duct-like or cyst-like structures. Recently, a few cases of rippled-pattern sebaceoma have been reported [2–4]. Histopathologic feature of the “rippled pattern” is originally reported as trichilemmal neoplasms (trichomatricoma, trichoblastoma) [5, 6]. Because sebaceoma can show a cribriform or reticular pattern as often seen in the trichoblastoma/trichoepithelioma, and trichoblastoma can present sebaceous differentiation, distinguishing sebaceoma from trichoblastoma with sebaceous differentiation is often extremely difficult [7]. However, we regarded the neoplasm in our case as sebaceoma, because histopathologic examination did not show the characteristic features observed in trichoblastoma (i.e., prominent fibrotic stroma, presence of follicular germs and rudimentary follicular papillae, or a palisading border in the neoplastic aggregations), but demonstrated the presence of mature sebocytes [7, 8]. There was no clinical history of nevus sebaceus and no such lesion was observed in the present case.

Dermoscopy is now widely used as a tool to diagnose many pigmented and nonpigmented cutaneous lesions. In the present case, dermoscopic examination demonstrated two discriminating features. One was clearly visualized yellowish homogeneous ovoid areas. This feature usually corresponds to dermal conglomerations of the cells with sebaceous differentiation. However, interestingly, this case histopathologically showed only limited area with sebaceous differentiation. The other feature was arborizing vessels at the peripheral area of the nodule. There were no arborizing vessels in the center of the tumor on dermoscopy or on clinical image as described in basal cell carcinoma [9]. Menzies et al. report that the specificity of this feature is 92% for diagnosis of pigmented basal cell carcinoma (BCC) [10]. It is also reported that arborizing vessels are seen characteristically in the cases of trichoblastoma [11]. Pluripotent stem cells in the folliculosebaceous-apocrine unit may give rise to follicular germinative cells and sebaceous germinative cells. Sebaceoma, trichoblastoma, and BCC, which are a malignant neoplasm of abnormal follicular germinative cells, are highly related to neoplasms embryologically [7, 8]. Therefore, it was considered that the feature of arborizing vessels could be commonly observed in the neoplasms derived from the folliculosebaceous unit.

In this report, we presented a case of rippled-pattern sebaceoma and described its intriguing dermoscopic features. 

## References

[1] Troy JL, Ackerman AB (1984). Sebaceoma. A distinctive benign neoplasm of adnexal epithelium differentiating toward sebaceous cells. *American Journal of Dermatopathology*.

[2] Kurokawa I, Nishimura K, Hakamada A (2007). Rippled-pattern sebaceoma with an immunohistochemical study of cytokeratins. *Journal of the European Academy of Dermatology and Venereology*.

[3] Kiyohara T, Kumakiri M, Kuwahara H, Saitoh A, Ansai S (2006). Rippled-pattern sebaceoma: a report of a lesion on the back with a review of the literature. *American Journal of Dermatopathology*.

[4] Misago N, Narisawa Y (2001). Rippled-pattern sebaceoma. *American Journal of Dermatopathology*.

[5] Akasaka T, Imamura Y, Mori Y, Iwasaki M, Kon S (1997). Trichoblastoma with Rippled-Pattern. *Journal of Dermatology*.

[6] Hashimoto K, Prince C, Kato I (1989). Rippled-pattern trichomatricoma. Histological, immunohistochemical and ultrastructural studies of an immature hair matrix tumor. *Journal of Cutaneous Pathology*.

[7] Misago N, Mihara I, Ansai S-I, Narisawa Y (2002). Sebaceoma and related neoplasms with sebaceous differentiation: a clinicopathologic study of 30 cases. *American Journal of Dermatopathology*.

[8] Misago N, Suse T, Uemura T, Narisawa Y (2004). Basal cell carcinoma with sebaceous differentiation. *American Journal of Dermatopathology*.

[9] Argenziano G, Zalaudek I, Corona R (2004). Vascular structures in skin tumors: a dermoscopy study. *Archives of Dermatology*.

[10] Menzies SW, Westerhoff K, Rabinovitz H, Kopf AW, McCarthy WH, Katz B (2000). Surface microscopy of pigmented basal cell carcinoma. *Archives of Dermatology*.

[11] Ohara K, Saida T, Ohara K, Tsuchida T (2003). Arborizing vessels. *Color Atlas of Dermoscopy*.

